# Effects of diabetes mellitus on the rate of carpal tunnel release in patients with carpal tunnel syndrome

**DOI:** 10.1038/s41598-021-95316-9

**Published:** 2021-08-04

**Authors:** Jaeyong Shin, Yong Wook Kim, Sang Chul Lee, Seung Nam Yang, Jee Suk Chang, Seo Yeon Yoon

**Affiliations:** 1 Department of Preventive Medicine and Public Health, Yonsei University College of Medicine, Seoul, Republic of Korea; 2 Department and Research Institute of Rehabilitation Medicine, Yonsei University College of Medicine, Seoul, Republic of Korea; 3 Department of Physical Medicine and Rehabilitation, Korea University Guro Hospital, Seoul, Republic of Korea; 4 Department of Radiation Oncology, Yonsei University College of Medicine, Seoul, Republic of Korea

**Keywords:** Risk factors, Peripheral neuropathies

## Abstract

The objective of this study was to evaluate the effects of diabetes mellitus (DM) on the rate of carpal tunnel release (CTR) using a large nationwide cohort in Korea and to identify risk factors, including comorbidities and socioeconomic status (SES), associated with CTR. Patients with a primary or secondary diagnosis of carpal tunnel syndrome (CTS; ICD-10 code: G560) were selected and divided into two groups according to the presence of DM. A Cox proportional hazard model was used to assess the rate of CTR between the two groups. To evaluate the influence of demographic factors, comorbidities, and SES on CTR, multivariate Cox proportional hazard regression models were used to adjust for confounding variables. In total, 12,419 patients with CTS were included in the study: 2487 in DM cohort and 9932 in non-DM cohort. DM duration was negatively related with the rate of CTR (HR = 0.89, 95% CI 0.87–0.91) in CTS patients with DM. The rate of CTR was decreased in patients with DM compared to those without DM in the unadjusted model; however, after adjusting for comorbidities, DM had no significant effect on the rate of CTR. Female sex (HR = 1.50, 95% CI 1.36–1.67) correlated with the rate of CTR, and an inverse relationship between the number of comorbidities and CTR was found (*p* < 0.001) irrespective of DM. Diabetic polyneuropathy (DPN) was not associated with CTR, and we did not find any factors correlating with CTR in DPN patients. We found that CTS patients with more comorbidities or combined with a longer duration of DM were undertreated in real-word practice. Actual outcomes of CTR in CTS patents with various comorbidities should be investigated in future studies for optimal management of CTS.

## Introduction

Various factors, such as repetitive wrist movement, hypothyroidism, obesity, diabetes mellitus (DM), and rheumatoid arthritis, have been suggested to be risk factors for Carpal tunnel syndrome (CTS)^1,2^. Recently, a meta-analysis of 25 studies suggested that both type 1 and type 2 diabetes are risk factors for CTS^3^. The prevalence of CTS is estimated to be approximately 3%^4^ in the general population and 14%^5^ in individuals with DM, increasing to 30%^5^ in the presence of diabetic polyneuropathy (DPN).

Treatment options for CTS include medication, splinting, injections, and carpal tunnel release (CTR)^6^. Several attempts, including rehabilitation and glycemic control, have been made to modify the natural history of CTS in diabetic patients, suggesting a potential role for improved glycemic control in the restoration of nerve function^7,8^. The effects of CTR in patients with both CTS and DM or DPN have been controversial. DM has been suggested to be associated with poorer outcomes after CTR in both short-term and long-term follow up^9,10^. However, several other studies have shown improvements in diabetic patients with CTS after CTR^11^, and some studies have found that CTR had similar effects in CTS patients irrespective of DM^12,13^. A recent meta-analysis showed the efficacy of CTR in relieving neurologic symptoms and restoring sensory deficits in patients with DPN^14^.

Previous studies about CTR in diabetic patients with CTS included a relatively small number of patients, and most previous work was conducted in western countries^12,13,15^. Differences in ethnic factors and healthcare management systems across countries might affect the rate of CTR. Comorbidities and socioeconomic status (SES) could also influence the rate of CTR in diabetic patients with CTS in real-world practice. Therefore, our aim in this study was to evaluate the effects of DM on CTR in patients with CTS using a large, nationwide, population-based cohort in Korea and to identify risk factors, including comorbidities and SES, associated with CTR. Additionally we assessed the effects of DPN on CTR and its risk factors for diabetic patients with CTS.

## Results

### Baseline characteristics of patients

**Figure 1:**
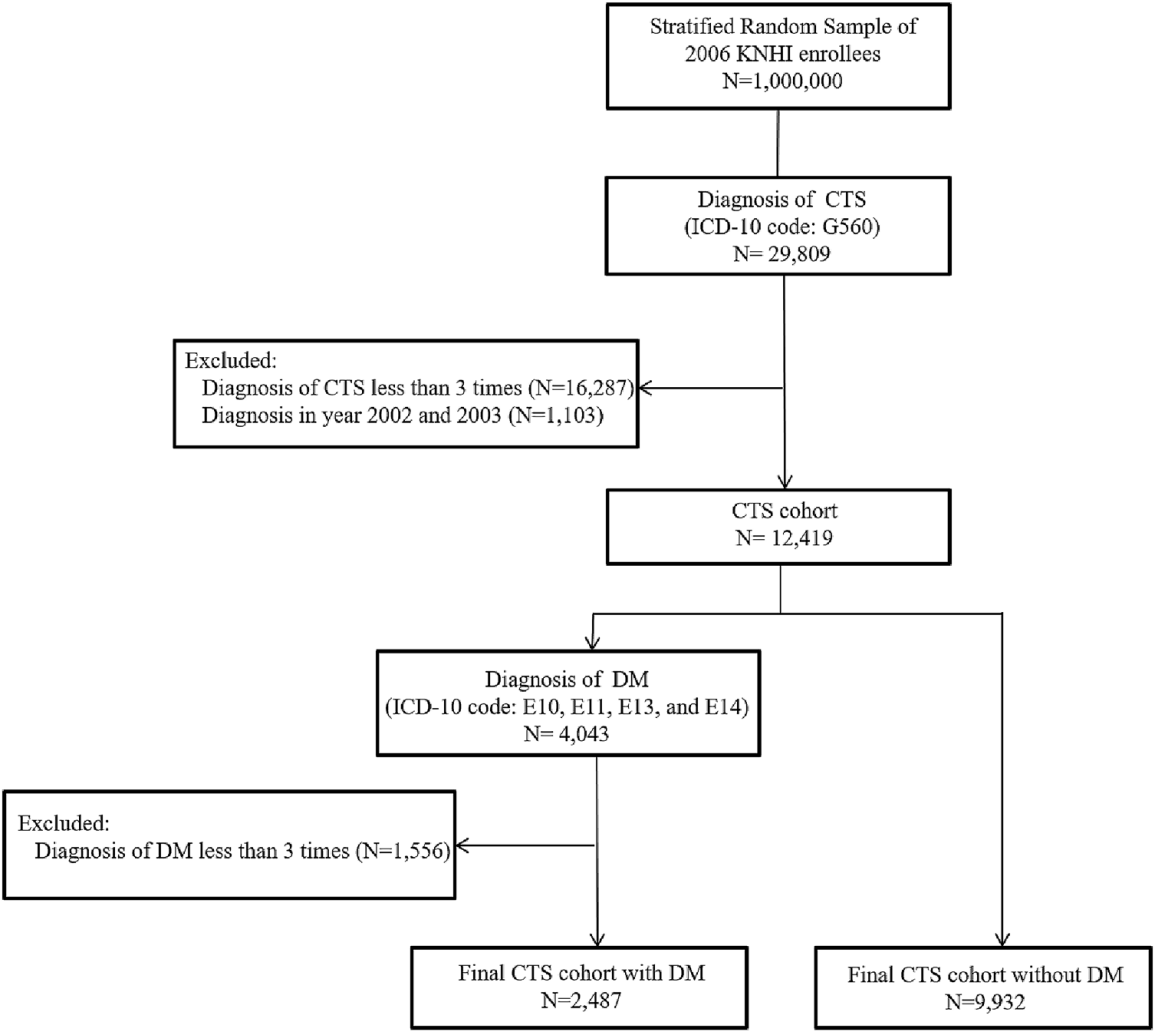
Flowchart for sample selection.

In total, 12,419 patients with CTS were included in this study: 2487 in the DM cohort and 9932 in the non-DM cohort (Fig. 1). Table 1 presents the demographic and medical characteristics of both groups, which differed significantly in age and sex. Males composed 24.90% of the DM group and only 18.44% of the non-DM group (*p* < 0.0001). Patients older than 60 years made up more than 50% of the DM group but only about 25% of the non-DM group (*p* < 0.0001). More patients in the DM group received medical aid insurance compared with the non-DM group (*p* < 0.0001). Patients with DM also had higher CCI scores and more comorbidities, including hypertension, hypothyroidism, and rheumatoid arthritis, than the non-DM group. Patients with DM had a lower rate of CTR than the non-DM group (*p* < 0.0001).

**Table 1:** Characteristics of the Study Populations with carpal tunnel syndrome (N = 12,419) according to presence of diabetes mellitus.

Variable	DM −		DM +		*p* value
	N	%	N	%	
Total	9932		2487		
**Carpal tunnel release**					
Yes	2509	25.26	530	21.31	< .001
No	7423	74.74	1957	78.69	
**Age group**					< .001
≤ 40	1084	10.91	60	2.41	
40–49	2262	22.77	268	10.78	
50–59	4129	41.57	874	35.14	
60–69	1637	16.48	763	30.68	
≥ 70	820	8.26	522	20.99	
**Sex**					< .001
Male	1831	18.44	619	24.90	
Female	8099	81.56	1867	75.10	
**Year of diagnosis**					< .001
2004–2007	2405	24.21	721	28.99	
2008–2011	3683	37.08	923	37.11	
2012–2015	3844	38.70	843	33.90	
**Regions**					0.022
Capital	1520	15.30	431	17.33	
Metropolitans	2633	26.51	615	24.73	
Rural	5779	58.19	1,441	57.94	
**Income level (quartiles)**					
Q1 (lowest)	2270	22.86	621	24.97	0.005
Q2	2568	25.86	564	22.68	
Q3	2876	28.96	721	28.99	
Q4 (highest)	2218	22.33	581	23.36	
**Insurance type**					< .001
NHI, employees	5876	59.17	1382	55.59	
NHI, self-employees	3663	36.89	910	36.60	
Medical aid	391	3.94	194	7.80	
**CCI**					< .001
0	849	8.55	105	4.22	
1	2282	22.98	337	13.55	
2	3171	31.93	594	23.88	
≥ 3	3630	36.55	1451	58.34	
**Other comorbidities**^**a**^					
Hypertension	3917	39.44	1928	77.52	< .001
Hypothyroidism	1052	10.59	326	13.11	< .001
Rheumatoid arthritis	2427	24.44	753	30.28	< .001

DM: diabetes mellitus; NHI: National Health Insurance.

### Factors associated with CTR in both groups

Table 2 shows the aHR for CTR according to DM based on the multivariate Cox proportional hazard models. Effects of DM duration on the rate of CTR was evaluated, and the results showed that DM duration was negatively related with CTR (HR = 0.89, 95% CI 0.87–0.91) in the DM group. Overall, female patients exhibited an increased risk for CTR compared with male patients (HR = 1.50, 95% CI 1.36–1.67), and the aHR was decreased in the DM cohort (HR = 1.35, 95% CI 1.09–1.68) and slightly increased in the non-DM cohort (HR = 1.54, 95% CI 1.37–1.74). As for comorbidities, an increased number of comorbidities significantly lowered the rate of CTR in patients with CTS in both the DM and non-DM groups (*p* < 0.0001). CTR correlated significantly with increasing age in the non-DM group; however, age and CTR did not correlate significantly in the DM group. Regarding residential area, patients living in rural areas showed an increased rate of CTR compared with patients living in the capital (HR = 1.15, 95% CI 1.02–1.29) only in the non-DM group. Income level and insurance type showed no significant influence on CTR in either group.

**Table 2:** Adjusted Hazard ratio for CTS release according to DM presence.

Variable	Total			DM −			DM +		
	HR	95% CI	*p* value	HR	95% CI	*p* value	HR	95% CI	*p* value
**Sex**									
Male	1.00						1.00		
Female	1.50	1.36–1.67	< .001	1.54	1.37–1.74	< .001	1.35	1.09–1.68	0.007
**Age group**									
≤ 40	1.00			1.00			1.00		
40–49	1.60	1.36–1.88	< .001	1.66	1.40–1.96	< .001	0.83	0.45–1.53	0.609
50–59	2.05	1.76–2.38	< .001	2.11	1.80–2.47	< .001	1.27	0.72–2.24	0.581
60–69	2.03	1.72–2.41	< .001	2.04	1.70–2.45	< .001	1.48	0.84–2.64	0.376
≥ 70	2.33	1.93–2.83	< .001	2.33	1.88–2.89	< .001	1.80	0.99–2.26	0.177
**Regions**									
Capital	1.00			1.00			1.00		
Metropolitans	1.06	0.94–1.19	0.348	1.05	0.97–1.20	0.426	1.08	0.83–1.44	0.500
Rural	1.15	1.04–1.27	0.009	1.15	1.02–1.29	0.020	1.02	0.90–1.46	0.263
**Income level (Quartiles)**									
Q1 (lowest)	1.00			1.00			1.00		
Q2	0.99	0.90–1.11	0.985	0.96	0.85–1.08	0.497	1.14	0.87–1.50	0.351
Q3	1.07	0.97–1.19	0.195	1.08	0.96–1.22	0.180	1.02	0.78–1.33	0.900
Q4 (highest)	0.98	0.87–1.10	0.707	0.97	0.85–1.10	0.593	1.02	0.77–1.36	0.900
**Insurance type**									
NHI, employees	1.00			1.00			1.00		
NHI, self-employees	1.03	0.96–1.11	0.439	1.01	0.93–1.10	0.792	1.08	0.90–1.30	0.403
Medical aid	0.95	0.78–1.16	0.620	0.91	0.72–1.15	0.420	1.02	0.70–1.50	0.903
**CCI**									
0	1.00								
1	0.64	0.57–0.72	< .001	0.63	0.56–0.72	< .001	0.82	0.57–1.18	0.035
2	0.49	0.44–0.56	< .001	0.50	0.44–0.57	< .001	0.54	0.38–0.77	< .001
≥ 3	0.34	0.29–0.38	< .001	0.33	0.29–0.38	< .001	0.42	0.30–0.58	< .001
DM duration							0.89	0.87–0.91	< .001

CCI: Charson comorbidity index.

### Rate of CTR depending on the presence of DM in CTS patients

The incidence rate of CTR in the DM group was 47 per 1000 person-years, whereas that in the non-DM group was 66 per 1000 person-years. Table 3 displays the unadjusted and adjusted HRs (aHRs) from univariate and multivariate Cox proportional hazard regression models for CTR during the 12-year follow-up period depending on the presence of DM. A decreased rate of CTR was observed in CTS patients with DM in the unadjusted model (model 1) overall (HR = 0.79, 95% CI 0.72–0.87) and among female patients (HR = 0.78, 95% CI 0.71–0.87). After adjusting for age and SES (model 2), those differences still existed overall (HR = 0.79, 95% CI 0.72–0.88) and in female patients (HR = 0.78, 95% CI 0.70–0.86). However, in model 3, which was further adjusted for comorbidities, the rate of CTR between the two groups did not differ significantly by the presence of DM. Among male patients, we found no significant differences in the rate of CTR between the two groups in the adjusted or unadjusted models.

**Table 3:** Multivariate cox regression analysis for carpal tunnel release in participants with carpal tunnel syndrome according to DM presence.

	Total			Male			Female		
		95% CI	*p* value		95% CI	*p* value		95% CI	*p* value
Diabetes mellitus (n)	2487			619			1867		
Person-year	11,095			2482			8602		
Carpal tunnel release (n)	530			106			424		
Incidence rate	47			43			49		
No	1 (ref)			1 (ref)			1 (ref)		
Yes									
Model 1^a^ HR	0.79	0.72–0.87	< .001	0.93	0.74–1.15	0.489	0.78	0.71–0.87	< .001
Model 2^b^ HR	0.79	0.72–0.88	< .001	0.92	0.74–1.16	0.482	0.78	0.70–0.86	< .001
Model 3^c^ HR	0.94	0.84–1.04	0.238	1.06	0.82–1.36	0.672	0.93	0.82–1.04	0.188

Incidence rate is the incidence of mortality per 1000 person-year.

^a^Model 1 : unadjusted.

^b^Model 2: adjusted for age and socioeconomic status (residential area, income level, and insurance type).

^c^Model 3: adjusted for age, socioeconomic status (residential area, income level, and insurance type), and co-morbidities (CCI, hypertension, hypothyroidism, rheumatoid arthritis).

### Effects of DPN on CTR in patients with CTS and DM

Among 2,487 CTS patients combined with DM, 370 (14.9%) patients were diagnosed with DPN. 78 (21.1%) DPN patients received CTR and 292 DPN patients (78.9%) did not received CTR (*p* = 0.907). Table 4 displays the unadjusted and adjusted HRs for CTR depending on the presence of DPN: DPN and CTR had no significant relationship in patients with DM and CTS. We also performed multivariate Cox proportional hazard regression models to find factors associated with CTR depending on the presence of DPN. After adjusting for confounding variables, female patients presented an increased risk for CTR compared with male patients (HR = 1.46, 95% CI 1.15–1.85), and comorbidities were inversely related to the rate of CTR only in the non-DPN group (*p* < 0.0001) (Table 5). We found no significant relationships between demographic, socioeconomic, and medical factors and CTR in the DPN group.

**Table 4:** Unadjusted and Adjusted Hazard Ratio for CTS release according DPN presence in participants with CTS and DM.

	DPN -	DPN +					
		Unadjusted			Adjusted		
		HR	95% CI	*p* value	HR	95% CI	*p* value
Total	1 (ref)	0.97	0.76–1.23	0.791	1.01	0.79–1.28	0.956
**Sex**							
Male	1 (ref)	1.44	0.88–2.36	0.151	1.29	0.78–2.14	0.319
Female	1 (ref)	0.86	0.65–1.13	0.285	0.92	0.70–1.22	0.564

**Table 5:** Adjusted Hazard ratio for CTS release stratified by DPN presence according in participants with CTS and DM.

Variable	DPN -			DPN +		
	HR	95% CI	*p* value	HR	95% CI	*p* value
**Sex**						
Male	1.00			1.00		
Female	1.46	1.15–1.85	0.002	0.85	0.50–1.46	0.553
**CCI**						
0	1.00					
1	0.67	0.46–0.98	0.038	0.71	0.19–2.68	0.613
2	0.47	0.33–0.68	< .001	0.25	0.07–0.98	0.057
≥ 3	0.31	0.21–0.44	< .001	0.51	0.15–1.77	0.288

Age and socioeconomic status were adjusted.

## Discussion

In this study, we analyzed 12,419 patients with CTS using information extracted from a nationwide cohort database (DB) of 1 million people. DM duration was negatively related with the rate of CTR in CTS patients combined with DM. On the other hand, there was no significant difference of the rate of CTR between CTS patients with and without DM after adjusting for comorbidities. An increased number of comorbidities significantly decreased the rate of CTR in both the DM and non-DM groups of CTS patients. Overall, female patients received more CTR than male patients. The rate of CTR increased gradually with patient age but only in the non-DM group; age did not influence CTR in the DM group. In the analysis of the DPN group, we found no factors correlated with CTR; not even female sex and comorbidities were related to CTR in the DPN group.

According to guidelines from the American Academy of Orthopedic Surgeons (AAOS), CTR is recommended as a grade A, level I treatment for CTS^16^. However, the AAOS CTS guidelines are based on studies that did not take patients’ medical comorbidities into account. Therefore, the AAOS treatment recommendations are considered inconclusive for patients with CTS and comorbidities such as DM. The influence of DM on the outcome of CTR has been controversial^9,11^, though some studies have suggested that CTR has similar effects in all CTS patients, irrespective of DM^12,13^. A previous meta-analysis found that patients with both CTS and DPN experienced beneficial effects from CTR in the form of relief from neurologic symptoms and restored sensory deficits^14^. These differences in the effects of CTR in diabetic patients with CTS might be caused by differences in study design, diagnostic criteria, or outcome variables. A diagnosis of CTS is usually based on clinical or electrophysiological findings, and the various labs in which tests were performed might have used different electrodiagnostic standards. Some studies focused on improvement in symptoms such as pain, numbness, or weakness^6,12^, whereas others evaluated improvement using electrophysiologic findings^14,15^.

In our results, DM duration showed negative relationship with the rate of CTR in CTS patients with combined DM. However, comparison for CTR between CTS patients with and without DM reveled somewhat different results. Patients with CTS and DM received less CTR in the unadjusted model; however, DM had no significant effect on CTR after adjusting for comorbidities. As the number of comorbidities increased, the rate of CTR decreased in all CTS patients, both with and without DM. In our results, patients with DM had more comorbidities than those without DM. We found that although patients with DM seemed to receive less surgery than those without DM, it was not the DM itself but the underlying comorbidities that truly affected the rate of CTR in CTS patients with DM.

Few reports have considered the effects of comorbidities on the outcomes of CTR. One previous study of the effect of comorbidities on CTR suggested that symptom severity and hand function improved significantly after CTR regardless of comorbidities^6^. In a previous study using a neural network to develop a prognostic model for CTR outcomes, hypertension was suggested to be related to poor outcomes^17^. However, no previous study has evaluated the relationship between the cumulative effects of comorbidities and CTR. Our results indicate that clinicians generally perform fewer CTR operations in patients with more comorbidities and in patients with a longer duration of DM. The actual effects of comorbidities, including DM, on the outcomes after CTR still remains uncertain. However, CTS patients with many comorbidities or combined with a longer duration of DM were undertreated in the real-word practice based on our results.

In this study, the number of CTS patients combined with DM is 2487, approximately 20% of total CTS patients. In pervious large cohort studies, the prevalence of DM in patients with CTS ranged from 3 to 5%^18–20^. In one retrospective cohort study in USA, among 1,795 patients with CTS, 254 patients (15%) were diagnosed with DM^21^. The CTS patients combined with DM have been reported as up be to 21% of all CTS patients in a prospective ultrasonographic and electrophysiologic study^2^, which is consistent with our finding. One possible reason for the relative high prevalence of DM in CTS patients in our study was that we included a DM diagnosis not only baseline but also during follow-up period. As the population ages, patients combined with CTS and various comorbidities including DM might become more prevalent, and the outcomes of CTR in these populations need further investigation for optimal management of CTS.

Overall, female sex was associated with the rate of CTR in both groups. Our findings support earlier studies that reported that female patients received CTR 3.0–5.5 times more than male patients^22^. Some putative reasons for the higher rate of CTR in female patients are that females have more intense subjective symptoms and are more prepared than males to undergo surgical treatment^22^.

According to the presence of DM, age and residential area showed different results: they were related to CTR in the non-DM group but showed no relationship with CTR in the DM group. Median nerve impairment has been suggested to become more severe with increasing age^23^. In a previous epidemiologic study, the rate of CTR peaked at 50–59 years in women and 60–69 years in men, suggesting a tendency for the elderly to avoid surgery^22^. However, in our analysis of recent data, the peak surgical rate was observed at or above 70 years. This implies that CTS patients with more severe symptoms get surgery irrespective of age, which is in line with other recent studies^24,25^. With regard to residential area, patients in rural areas received more CTR than those in the capital region, which might be related to their occupations, such as agriculture which is strenuous on wrist. However, among patients with both CTS and DM, age and residential area did not influence the rate of CTR, so it seems that overuse and a hand-intensive workload are not related to the rate of CTR in patients with DM. In terms of SES, we found no effects on the rate of CTR.

In patients with both CTS and DPN, we found no factors associated with CTR; not even female sex and comorbidities affected the rate of CTR in this subpopulation. It was previously suggested that DPN, a length-dependent axonopathy, makes the median nerve more susceptible to CTS^5^ The severity of median nerve impairment and the symptoms of neuropathy might affect the rate of CTR irrespective of sex or comorbidities. Studies are warranted to determine the factors that predict the effects of CTR in DPN patients.

This study has several limitations. First, the diagnoses of DM, DPN, and CTS are based entirely on ICD codes instead of a standardized electrodiagnostic process. This was a retrospective study using NHIS cohort data, so we could not get the electrophysiologic findings. However, the disease diagnoses are valid and relatively reliable because the NHIS program conducts regular cross-checking by reviewing chart records and laboratory results to prevent miscoding or inaccurate medical claims data. Second, some information including the side of CTR, lifestyle factors, medication, or certain laboratory result such as HbA1c were not considered in the analyses, which could affect the results. Instead, we tried to adjust other various factors including SES and comorbidities available in our DB. Third, in this epidemiologic study, we sought factors related to CTR, including DM, DPN, comorbidities, and SES, but we could not elucidate the effects of the various factors on the outcomes after CTR. Future studies are needed that will investigate predictive factors for CTR based on the variables in this study.

In conclusion, we evaluated the effects of DM on CTR in patients with CTS using a large, nationwide, population-based cohort in Korea and found that although patients with DM seemed to receive less surgery than those without DM, it was not the DM itself but the underlying comorbidities that truly affected the rate of CTR in CTS patients with DM. On the other hand, in CTS patients with DM, as the DM duration increased the rate of CTR decreased. As for DPN group, we found no factors correlated with CTR; not even female sex and comorbidities were related to CTR in the DPN group. In this study, we found that CTS patients with more comorbidities or combined with a longer duration of DM were undertreated in real- word practice. Based on our results, outcomes of CTR in CTS patents with various comorbidities should be investigated in future studies for optimal management of CTS.

## Materials and methods

### Data source

The Korean National Health Insurance Service (NHIS), a single-payer system, is mandatory for all residents of Korea, and nearly all of the data in the health system are centralized in large DB. This nationwide, population-based cohort study was conducted using Korean National Health Insurance Service (NHIS) – National Sample Cohort (NSC) data. NHIS NSC DB is an approximately 2% random sample (n = 1,000,000) of all citizens stratified according to age, sex, income level and regional area in 2006. Data were produced by the NHIS using a systematic sampling method for the purpose of research. Random sampling was used based on 2142 strata (2 categories for sex, 17 categories for age group, 21 categories for income, and 3 categories for regional area). The data include a unique anonymous number for each patient and summarize age, sex, type of insurance, a list of diagnoses according to the International Classification of Diseases (ICD-10), and medical costs claimed. The NHIS DB represents the entire Korean population; therefore, it can be used for nationwide population-based studies of various diseases^26–28^. Also, it has been shown that this database provides reliable estimates of the real prevalence of various diseases in Korea^29–31^. This study was approved by the Institutional Review Board of Bundang Jesaeng General Hospital, which waived the need for informed consent. All methods were carried out in accordance with relevant guidelines and regulations.

### Study population

From the DB, we first selected patients with a primary or secondary diagnosis of CTS (ICD-10 code: G560) between 2002 and 2015. To ensure diagnostic validity, we included only individuals who visited clinics and received a diagnosis of CTS more than three times^32–35^. We excluded individuals who were diagnosed with CTS in 2002 and 2003 to ensure that the CTS group included only subjects with new episodes. Among the patients diagnosed with CTS, we selected patients who also had a primary of secondary diagnosis of DM (ICD-10 codes: E10, E11, E13, and E14) to divide our cohort into two groups according to the presence of DM. For patients who received CTR, DM at baseline and during the follow-up period to the date of CTR was selected, and for patients who did not receive CTR, DM at baseline and until end of follow-up period was selected. CTS patients who received surgical procedures coded as N0931 (simple reconstruction of tendon and ligament) or N0932 (complex reconstruction of tendon and ligament) were considered to have received CTR.

### Other variables

Age was categorized into 5 groups: ≤ 40 years, 40–49 years, 50–59 years, 60–69 years, and ≥ 70 years. We divided residential areas into capital, metropolitan, and rural areas. Korea contains six metropolitan cities with populations exceeding 1 million people: Busan, Incheon, Daejeon, Daegu, Gwangju, and Ulsan. Patients living in those cities were categorized as living in a ‘metropolitan’ area. Patients living in areas other than Seoul, the capital of Korea, or other metropolitan cities were classified as living in ‘rural’ areas. The NHIS premium was used as a proxy measure of precise income because it is proportional to monthly income, including earnings and capital gains. The income deciles of enrolled subjects were categorized into four categories (Q1: all medical aid enrollees + 0–20 percentiles of NHIS enrollees, Q2: 21–50 percentiles of NHIS enrollees, Q3: 51–80 percentiles of NHIS enrollees, Q4: 81–100 percentiles of NHI enrollees).

DPN was defined as ICD-10 codes E1041, E1141, E1341, E1441, G628, G629, G632, G633, and G638. Comorbidity was defined using the Charlson Comorbidity Index (CCI). The CCI is the most widely used morbidity index, which has been used to represent various comorbidities in previous epidemiologic studies^36,37^. Its validity has been confirmed by comparing with other morbidity indices, and it is applicable to the longitudinal study design^38^. Diseases in the CCI are congestive heart failure, myocardial infarction, cerebrovascular disease, peripheral vascular disease, connective tissue disease, chronic lung disease, ulcer, chronic liver disease, severe liver disease, dementia, diabetes, hemiplegia, moderate or severe kidney disease, tumor, leukemia, lymphoma, moderate or metastatic solid tumor, and acquired immunodeficiency syndrome. In this study, due to data missing from the NHIS-NSC DB, we could not include diagnoses related to dementia, tumor, or acquired immunodeficiency syndrome. Because DM was the main independent variable in this study, we also excluded DM from the CCI scoring. For the calculation of CCI score, each disease was scored from 0 to 3 points, and the greater score presents more comorbid condition. Then we categorized CCI scores into four ranges: 0, 1, 2, and 3 or more. In addition to the CCI, we extracted several diseases that could be related to DM or CTS using the ICD-10 codes for hypertension, hypothyroidism, and rheumatoid arthritis.

### Statistical analyses

The baseline categorical variables of both groups are expressed as numbers and percentages. The chi-squared test was used to compare the distributions of baseline demographic characteristics and selected comorbidities between patients with and without DM. Univariate and multivariate Cox proportional hazard regression analyses were performed to identify hazards associated with CTR depending on the presence of DM or DPN in terms of hazards ratios (HRs) and 95% confidence intervals (CIs). To evaluate the influence of demographic factors, comorbidities, and SES in both groups (with/without DM), multivariate Cox proportional hazard regression models were used after adjusting for confounding variables. We used SAS software version 9.4 (SAS Institute Inc, Cary, NC, USA) to perform all statistical analyses. *P* < 0.05 was considered statistically significant.

SAS System for Windows statistical software, version 9.4 (SAS Institute Inc, Cary, NC, USA) was used to perform all statistical analyses.
