# Author Correction: Effects of diabetes mellitus on the rate of carpal tunnel release in patients with carpal tunnel syndrome

**DOI:** 10.1038/s41598-021-96729-2

**Published:** 2021-08-19

**Authors:** Jaeyong Shin, Yong Wook Kim, Sang Chul Lee, Seung Nam Yang, Jee Suk Chang, Seo Yeon Yoon

**Affiliations:** 1 Department of Preventive Medicine and Public Health, Yonsei University College of Medicine, Seoul, Republic of Korea; 2 Department and Research Institute of Rehabilitation Medicine, Yonsei University College of Medicine, Seoul, Republic of Korea; 3 Department of Physical Medicine and Rehabilitation, Korea University Guro Hospital, Seoul, Republic of Korea; 4 Department of Radiation Oncology, Yonsei University College of Medicine, Seoul, Republic of Korea

Correction to: *Scientific Reports* 10.1038/s41598-021-95316-9, published online 04 August 2021

The Funding section in the original version of this Article was omitted. The Funding section now reads:

“This study was supported by a faculty research grant of Yonsei University College of Medicine for (6-2021-0158)."

The original Article has been corrected.

